# Our Sick and Wounded Home Again

**Published:** 1914-09-12

**Authors:** 


### HOSPITALS AND THE WAR.

### OUR SICK AND WOUNDED HOME AGAIN.

### WHERE THEY ARE UNDER TREATMENT.

It has been no easy task to set about making arrangements for the reception of our sailors and soldiers suffering from sickness and wounds received during the greatest war the world has probably ever seen. The total casualties among the soldiers at the seat of war, including the missing already reported, approach 20,000. Of these, we are glad to note that a large number are included under "missing," because there is reason to hope that a good proportion of them may be alive and well, and that those who are not prisoners may have rejoined or are in process of rejoining the Colours. Still, the number of wounded already is large, and that number must be very greatly increased, we are afraid, as the result of the great battles which are now proceeding in France between the Germans and the Allies. It follows that one essential to the successful and comfortable housing of the wounded on reaching this country is that the actual number of hospital beds available shall be adequate. It is, therefore, a matter of grave importance that an official announcement should be made without delay as to the number of beds actually available at the present time for the reception of wounded and sick men from the front under all heads. That is, the number of beds provided in military hospitals, Territorial hospitals, voluntary hospitals of all kinds, in homes or special hospitals approved by the British Red Cross Society, and in semi-convalescent institutions.

Judging by the reports received of the character of the injuries of most of the men already admitted to the voluntary hospitals, we may hope that the majority of cases will be relatively slight, and that the actual days' residence of the patients in the hospitals will be relatively few. This hope is confirmed by the fact that a large number of the patients admitted to the London Hospital on the 30th ultimo were discharged to convalescent homes or elsewhere on the 7th instant.

One point which seems worthy of special attention is the organisation of an adequate ambulance and motor service, which would enable the wounded and sick to be removed from the railway stations to the hospitals and other institutions where beds await them with the minimum of risk and discomfort to the injured. It is not right, nor ought it to be permitted, that our warriors who come back from the seat of war suffering from wounds or sickness, after long and wearing travel by land and sea, should have any but the best service which the most efficient organisation and skilled knowledge can provide to ease the sufferings of transit and the risks of making serious wounds or fractures worse by inexperienced handling or the use of improper or ill-adapted vehicles. The War Office is fully alive to the importance of what we here urge. Every effort has been made to perfect the arrangements. We have good reason to believe that whenever trains full of wounded may arrive at a station an adequate force of helpers and a sufficient number of motors and ambulances will be found prepared in readiness to take them as speedily as possible to the hospital, where they will be provided with the best of treatment and nursing and every possible comfort.

#### Motor Ambulances for the Front.

It is with great gratification, too, that we are in a position to report that the Metropolitan Asylums Board, at the request of the General of the Southern Command, have stationed a motor ambulance, with driver and attendant, at Southampton Docks for the conveyance of the sick to Netley Hospital. They have also at the request of the military authorities undertaken to supply, as required, ambulances for the conveyance of considerable numbers of sick soldiers to the several military hospitals in and near London. The Board at present have six motor ambulances almost ready for use; a further three chassis, which were ready for delivery, have been appropriated by the military authorities, who were not informed that they belonged to the Board, and so sent abroad. Having regard to the calls that have been made on the Board's service, and to the probability of having to meet further demands, the Board have decided to purchase ten more motor-ambulance chassis.

